# Non-cell autonomous downregulation of the purinergic receptor P2Y1R promotes neuroprotection after ischemic injury

**DOI:** 10.3389/fncel.2026.1790325

**Published:** 2026-04-21

**Authors:** Gabrielle Spagnuolo, Lorraine Iacovitti

**Affiliations:** 1 Department of Neuroscience, Farber Institute for Neurosciences, Thomas Jefferson University, Philadelphia, PA, United States; 2 Department of Neurology, Farber Institute for Neurosciences, Thomas Jefferson University, Philadelphia, PA, United States; 3 Department of Neurosurgery, Farber Institute for Neurosciences, Thomas Jefferson University, Philadelphia, PA, United States

**Keywords:** cerebral ischemia, glia, non-cell autonomous mechanisms, oxygen–glucose deprivation, purinergic receptors

## Abstract

Current ischemic stroke treatments largely focus on exogenous means of neural repair, with endogenous mechanisms being less understood. Here, we examine the cellular and molecular foundation of an endogenous neuroprotective mechanism using the *in vitro* stroke model oxygen–glucose deprivation (OGD). We demonstrate that after OGD, dying cortical neurons release ATP to activate microglia. There is a simultaneous increase in microglial release of B-NGF and IL-2, increased TrkA receptor expression on astrocytes, and a consequent downregulation in astrocyte P2Y1 receptors (P2Y1R), resulting in a decline in neuronal intracellular calcium levels and enhanced neuronal survival. This neuroprotective effect is mimicked when P2Y1R expression is directly knocked out in astrocytes or when exogenous microglial activators IL2 or NGF are added in place of microglia. Conversely, these neuroprotective effects are prevented by blockade of microglial activation or inhibition of TrkA or IL-2 receptors. Pharmacological buffering of intracellular Ca^2+^ with BAPTA-AM recapitulated the neuroprotective effect, whereas NMDA receptor blockade with Dizocilpine maleate did not, indicating that neuronal survival is mediated by reduced intracellular Ca^2+^ accumulation through an NMDA receptor–independent mechanism. Together, these results suggest the downregulation of P2Y1R in astrocytes by activated microglia is a critical endogenous neuroprotective mechanism after ischemic injury. By understanding these inherent non-cell autonomous mechanisms and their molecular mediators, it may be possible to improve intrinsic neuroprotection and recovery from stroke.

## 1 Introduction

A central question in ischemic stroke research is how to protect neurons from injury and death. Until recently, much of the focus has centered on intrinsic ways to target neurons directly, but this overlooks the important contribution other brain cell types and non-cell autonomous mechanisms (i.e., the ability of genes to affect cells outside of the cell where the genes are present) play in neuronal recovery (Biernaskie et al., 2004; Blanco-Suarez and Allen, 2022; Burda and Sofroniew, 2014; Dobkin and Carmichael, 2016; Heiss, 2012; Krakauer et al., 2012). Through studying cells such as astrocytes, microglia, and their interactions, it may be possible to capitalize on repair processes already present in the brain and potentially uncover new therapies for stroke. Purinergic receptors, particularly P2Y1R signaling, have been implicated in a number of important roles in the brain, including mediating cellular damage after injury (Butt, 2011; Verkhratsky and Toescu, 2006). While P2Y1R downregulation has previously been implicated in neuronal rescue from ischemic stroke (Kuboyama et al., 2011) and astrocyte-driven neuronal hyperexcitability (Shigetomi et al., 2024), the molecular correlates at critical steps in the neuroprotective process remain an enigma and therefore were the object of our study.

During stroke, dying cells release increased ATP and other purines into the extracellular space due to stress, activating their respective purinergic receptors on neurons and glia (Burnstock, 2016; Burnstock and Knight, 2004). One subfamily of purinergic receptors, the P2Y1 receptors (P2Y1Rs) have been implicated in several processes that contribute to the progression of neurological disorders. Of significance, P2Y1R activation can facilitate glutamate release through its mediation of calcium signaling and contribute to excitotoxicity and synaptic dysfunction in neurons (Burnstock and Knight, 2004).

Conversely, previous work utilizing the ischemic-like injury model oxygen–glucose deprivation (OGD) on tissue slices found that administering a P2Y1R antagonist prior to OGD prevented synaptic failure in the dentate gyrus (Maraula et al., 2014). As this study induced a global blockade of P2Y1R, it is impossible to know if the neuroprotective effects of P2Y1R antagonism were due to direct antagonism of P2Y1R in neurons or a surrounding cell type. As glia can act through non-cell autonomous mechanisms (Miller et al., 2020) and P2Y1R is found on all cell types in the CNS (Burnstock and Knight, 2004), it is important to understand if this mechanism of neuronal survival represents a cell or non-cell autonomous effect.

Further emphasizing the significance of non-cell autonomous effects in P2Y1R-mediated neuroprotection, activated microglia, which are normally viewed as pro-inflammatory, were counter-intuitively shown to convert astrocytes into a neuroprotective phenotype after injury through the downregulation of P2Y1R specifically on astrocytes in a model of traumatic brain injury (Shinozaki et al., 2017). However, the mechanism through which this occurred was studied in the context of glial scarring. While recent work has shown that P2Y1R upregulation in astrocytes during injury promotes neuronal hyperexcitability (Shigetomi et al., 2024), potential mechanisms of P2Y1R-mediated neuroprotection through downregulation of the receptor are not well understood.

The non-cell autonomous mechanism through which microglia may convert astrocytes into a neuroprotective phenotype through downregulation of astrocyte P2Y1R has not been thoroughly studied in models of ischemic stroke. Therefore, the focus of our work is to further understand the role of individual cell types (neurons, astrocytes, and microglia) and their molecular crosstalk in stroke recovery utilizing an *in vitro* model of stroke. Our hope is that modulation of the P2Y1R receptor in a cell-type specific manner may eventually reveal its therapeutic potential to facilitate recovery in stroke patients.

## 2 Materials and methods

### 2.1 Neuronal culture preparation

Timed pregnant Sprague–Dawley rats were purchased from Envigo (002) or timed pregnant C57BL/6 mice from Jackson Labs (Stock #000664) unless otherwise stated. On embryonic day 14.5 (E14.5) or E12.5, timed pregnant adult females were euthanized by exposing the animals to 30–70% gradual displacement rate of CO₂ until cessation of breathing occurred in accordance with Thomas Jefferson University IACUC protocols 01499 and 00127. Thoracotomy was performed and embryos were harvested and placed in ice cold DPBS (Gibco #14190–144). As previously described (Kostuk et al., 2019), embryos were visualized under a dissection microscope (Nikon SMZ1500; adjustable 1–11.5x objective) and cortical tissues collected and placed in 2.5 mL of enzymatic dissociation solution containing 5 mg DNase I (Sigma #10104159001), 5 mg Papain (Sigma #10108014001), and 50 mg L-cysteine (Fisher AAJ6374522) for up to 30 min. Solution was gently agitated every 5 min to aid in dissociation. Tissue was mechanically dissociated by gently triturating 8–10 times using a 1 mL pipettor with low retention tips. Cells were then pelleted for 5 min at 1000 rpm, re-suspended in culture media and counted on an automated hemocytometer (Countess FLII Invitrogen). Cells were plated on wells pre-coated with poly-L-lysine (PLL, 0.5 mg/mL) at a density per well of 5×10^5^ cells on 6-well plates, 1×10^5^ cells on 24-well plates, or 5×10^4^ cells on 96-well plates.

### 2.2 Glial culture preparation

Homogeneous cultures of cortical astrocytes and microglia were prepared according to previously published methods (Kostuk et al., 2019; Schildge et al., 2013; Weinstein, 2001). For rat cultures, postnatal day 1–5 Sprague–Dawley pups were anesthetized on ice and quickly decapitated in accordance with Thomas Jefferson University IACUC protocol 01499. Brains were removed and placed in ice cold DPBS. Brains were visualized under a dissection microscope (Nikon SMZ1500). Whole cortices were collected and dissected into smaller pieces prior to enzymatic digestion. Tissues were then subjected to enzymatic digestion using a Trypsin (0.1%)/DNAse I (0.5 mg/mL) mixture for 30 min at 37 °C, with gentle agitation every 5 min to aid in dissociation. The supernatant enzyme was carefully aspirated and tissues washed twice in DPBS. Tissues were then mechanically dissociated in 0.5 mg/mL DNAse I and gently triturated 8–10 times. Cells were pelleted for 5 min at 1000 rpm, re-suspended in media, and plated on T75 tissue culture flasks. Cultures were maintained for 2–4 weeks until confluent with media changes every other day.

For mouse cultures, the same procedure was followed in accordance with Thomas Jefferson University IACUC protocol 00127 using C57B6/J mice (unless otherwise noted) with the exception that trypsin (0.05%)/DNAse I (0.5 mg/mL) was used as the enzymatic digestive solution.

Once confluent, flasks were shaken at 200 RPM for 24 h to separate astrocytes (adherent) and microglia (non-adherent) and the non-adherent cells collected. The cell suspension was passed through a 70-mm cell strainer prior to centrifugation at 1000 rpm for 5 min. The cells were re-suspended in culture media for experimental use.

### 2.3 Transgenic mouse cells

For mouse studies utilizing P2Y1R knockout (KO) mice, mice (Fabre et al., 1999) were purchased from Jackson Labs (Stock #009131). Heterozygotes were bred (Protocol 000127) to produce a homozygous colony and confirmed through genotyping according to Jackson Labs protocols prior to culture as described above.

Homozygous TrkA^F592A^ mice (Chen et al., 2005) were generously donated by Ryan Tomlinson at Thomas Jefferson University. Cells were cultured as described previously and then treated with 100 nM 1NMPP1, a PP1-analog, throughout the injury period to inhibit TrkA signaling specifically in TrkA^F592A^ cells.

Mouse PCR primers for genotyping:

**Table 1:** 

oIMR9708 Common Fwd	TCTTCTACTCTGGCACTGGGACTC
oIMR9710 Mutant Rev	GCTTCCTCGTGCTTTACGGTAT
WT ms *P2ry1* Rev.	CTAGGATCTCGTGCCTTCACAAAC
TRKA-F592A-Fwd	5’ AACAGTTTTGAGCATTTTCTATTGTTTAAAAG 3’
TRKA-F592A-Rev	5’ CAAAGAAAACAGAAGAAAAATAATACATGAAG 3’

### 2.4 Co-culture studies: astrocytes and microglia

Cortical astrocytes were isolated and cultured as described above. Cells were lifted from their plate, dissociated to a single cell suspension, quantified, and plated onto appropriate tissue culture plates at a density per well of 1.5×10^6^ cells on 6-well plates, 3×10^5^ cells on 24-well plates, or 1.5×10^5^ cells on 96-well plates. Cells were allowed to adhere and grow for 24 h prior to further experimentation. Microglia were added to a transwell insert (Corning) at a density per well of 2.5×10^5^ cells on 6-well plates, 5×10^4^ cells on 24-well plates, or 2.5×10^4^ cells on 96-well plates. This allowed the cells to share media but not directly interact. The following day, cells were used for experimental purposes.

### 2.5 Co-culture studies: neurons, astrocytes, and microglia

Cortical neurons were isolated and plated as described above on appropriate PLL-coated wells. Neurons were allowed to adhere and mature for 7 days. After this time, cortical astrocytes were lifted, dissociated to a single cell suspension, quantified, and plated on a transwell insert sitting above the neurons at a density per well of 1.5 × 10^6^ cells on 6-well plates, 3 × 10^5^ cells on 24-well plates, or 1.5 × 10^5^ cells on 96-well plates, sharing media. Microglia were added to the well with the neurons at a density per well of 2.5 × 10^5^ cells on 6-well plates, 5 × 10^4^ cells on 24-well plates, or 2.5 × 10^4^ cells on 96-well plates, allowing astrocytes to be analyzed separate from the other cell types. Cells were allowed to adhere and grow for an additional 24 h prior to further experimentation.

### 2.6 Oxygen–glucose deprivation

Similar to previous studies (Guo et al., 2023; Qin et al., 2024; Lin et al., 2016), cells were plated as described above. OGD media (glucose/phenol-free DMEM + 1% pen strep) was deoxygenated by gassing with 95% nitrogen and 5% CO_2_ for 15 min. Cell cultures were washed twice with PBS and then placed in OGD or normoxic media (phenol-free DMEM + 1% pen strep).

For experiments involving inhibition of microglial activation, cells were treated with either 100uM minocycline (Fisher AAJ66429ME) or 5uM Pexidartinib (MedChemExpress LLC HY16749) by adding it into the OGD media prior to putting on cells (Yrjänheikki et al., 1998; Van Zeller et al., 2022).

For experiments involving inhibition of the B-NGF receptor TrkA, 2.0 nM (manufacturer’s recommendation) of the specific TrkA competitive antagonist GW 441756 (Tocris 2,238) was added to the OGD media at the stated concentrations.

For experiments involving inhibition of the IL-2 receptor IL-2R, the inhibitor Basiliximab (Fisher 50–313-7348) was added to the OGD media at 3.5 nM based on manufacturer’s recommendation for blockade of receptor-ligand interaction.

For experiments involving blockade of Ca^2+^ flux, 2.5 nM of the non-competitive NMDA receptor antagonist Dizocilpine maleate (Thermo Fisher J63917. MF) was added to cultures during and after OGD.

For experiments utilizing the Ca^2+^ chelator, 5.0uM BAPTA (Thermo Fisher B1205) was added to cultures before and during OGD.

The OGD cultures were then put in a sealed chamber (Billups-Rothenberg, Inc., Del Mar, CA, United States), flushed with 95% nitrogen and 5% CO_2_ for 6 min at a flow rate of 20 L/min and incubated for 2 h or 6 h at 37 °C. Cells then had 4.5 mM glucose added back (to match the concentration in the normoxic group) and returned to a regular 5% CO_2_ incubator for 24 h. After this time, cells were processed for downstream analysis.

### 2.7 Injury from exogenous ATP

Primary cortical astrocytes and microglia were plated as described above. Cultures were washed 2x with PBS and all placed in normoxic media. For cultures undergoing injury, 10^−10^ mol exogenous ATP (Fisher FERR0441) was added to the media for 24 h. After 24 h, cells were harvested for downstream analysis.

### 2.8 Viability assays

After the 24 h reperfusion period of OGD samples, cell viability was analyzed using LIVE/DEAD Cell Imaging Kit (488/570, Molecular Probes, Life Technologies Corp., CA, United States). Cells were imaged immediately at 20x (high content fluorescent microscope Keyance BZ-X710) and cell count quantified. Viability was also assessed with the Cell Proliferation Kit I (MTT) (Sigma 11,465,007,001) and read on a MR9600-T SmartReader (Accuris Instruments).

### 2.9 RNA isolation and cDNA synthesis

Total RNA was isolated directly from freshly collected cells in TRIzol (Invitrogen), a modification of the guanidine isothiocyanate-phenol-chloroform extraction method. cDNA was synthesized by using at least 250 ng total RNA in a 20 μL reaction with Superscript IV (Invitrogen) and oligo (dT)12–18 (Invitrogen). One microliter of Rnase Out (Invitrogen) was added to each reaction tube, and the tubes were incubated for 20 min at 37 °C before proceeding to real-time PCR.

### 2.10 Real-time PCR analysis

Real-time PCR was carried out on the 7,500 Real Time PCR System using SYBR green PCR master mix (both from Applied Biosystems). GAPDH was used as an internal control. PCR analyses were conducted in triplicate for each sample. The reaction mix consisted of 6.35 ng cDNA, 0.5 μM forward and reverse primer mix, 1x SYBR green PCR master mix. Reactions were run according to manufacturer protocols for at least 40 cycles. Data were analyzed using the ratiometric ΔΔCT method, and the mean relative mRNA expression for each sample was reported.

Rat qPCR primer sequences:

**Table 2:** 

GAPDH Fwd	CTCAGTTGCTGAGGAGTCCC
GAPDH Rev.	ATTCGAGAGAAGGGAGGGCT
P2Y1R Fwd	GAACAGCCGAACTACTTGGAC
P2Y1R Rev.	CTTGTCGGCTTCATGAACCTC
TrkA Fwd	GGTCTTTCTTGCTGAGTGCTAC
TrkA Rev.	GCTGAAAGTCCTGCCGAGCATT

### 2.11 Western blot

Similar to previous studies (Cai et al., 2013; Lin et al., 2016; Zhao et al., 2024; Chen et al., 2023), cells were lysed in RIPA buffer (Thermo Scientific 89,901) with HALT protease (Thermo Scientific 78,430) and phosphatase (Thermo Scientific 78,420) inhibitor cocktails. Protein concentration was determined using the BIORAD DCtm Protein Assay (BIORAD 5000111) according to manufacturer’s instructions. Protein samples of 20 μg total protein were loaded in duplicate into NuPAGE 4–12% Bis-Tris protein gels (Invitrogen NP0335BOX) and run in NuPAGE MES Buffer (Invitrogen NP0002) with Novex Sharp Pre-stained protein standard (Invitrogen LC5800). Gels were transferred to nitrocellulose membranes using the Invitrogen iBlot transfer system. Membranes were then blocked in 5% non-fat dry milk in tris-buffered saline with 0.1% Tween. Blots were subsequently incubated with appropriate primary antibodies (GAPDH: Fitzgerald Industries International 10R-G109a; P2Y1R: Invitrogen MA5-31814; TrkA: Invitrogen MA5-32123 overnight at 4 °C). Blots were then incubated with species appropriate HRP-conjugated secondary antibodies (Thermo Scientific 31,460 or Thermo Scientific 31,431) used at manufacturer’s recommended concentrations for 1 h at room temperature. Bands were visualized using Thermo SuperSignal West Pico PLUS Chemiluminescent Substrate (Thermo Scientific 34,580) on the Bio-Rad ChemiDoc. Densitometric analyses were performed using the NIH Image program (ImageJ software), and the ratio between the protein and the corresponding loading control was calculated.

### 2.12 ATP assay

The amount of ATP released from cells after OGD was assessed using the ENLITEN ATP Assay system (Promega). ATP was then exogenously added to samples plated as described above in “Co-culture studies: astrocytes and microglia.”

### 2.13 Cytokine assay

The relative amount of cytokines released after OGD were measured using the Rat Cytokine Array C2 (RayBiotech AAR-CYT-2-8). Based on these levels, appropriate concentrations of cytokines were exogenously added to samples as described.

### 2.14 Exogenous addition of cytokines and growth factors

Exogenous cytokines and growth factors were added to astrocytes for 24 h, after which cells were harvested for downstream analysis. Reagents were purchased and added to cells in the dosages as follows: Activin A (Stem Cell Technologies 78,132; 100 ng/mL), Agrin (R&D Systems 550-AG; 100 ng/mL), B-NGF (R&D Systems; 556-NG; 1 ng/mL), B7-2/CD86 (R&D Systems 1,340-B2; 5ug/mL), CINC-1 (R&D Systems 515-CN; 10 ng/mL), Fas ligand (R&D Systems 2,159-FA; 20 ng/mL), Fractalkine (R&D Systems 537-FT; 10 ng/mL), ICAM-1 (R&D Systems 583-IC; 10 ug/mL), IFN-g (R&D Systems; 585-IF; 1 ng/mL), IL-10 (R&D Systems 522-RLB; 1 ng/mL), IL-1a (R&D Systems 500-RL; 1 ng/mL), IL-1b (R&D Systems 401-ML; 1 ng/mL), IL-2 (R&D Systems 502-RL; 1 ng/mL), IL-6 (R&D Systems 406-ML; 1 ng/mL), TNF-a (R&D Systems 410-MT; 1 ng/mL), VEGF (R&D Systems 493-MV; 5 ng/mL).

### 2.15 Calcium assay

Neuronal intracellular calcium concentrations were assessed using a Calcium Assay Kit (Abcam ab102505) according to manufacturer’s protocol.

### 2.16 Statistical analysis

All data are presented as the mean ± SEM. Samples were always run in technical duplicates or triplicates with at least 3 independent biological replicates. GraphPad was used for all statistical analysis. The statistical significance of the mean difference was calculated using a one- or two-way ANOVA with appropriate *post hoc* analysis of significance or a two-tailed Student’s *t*-test. A *p*-value ≤ 0.05 was considered significant.

## 3 Results

### 3.1 Neuronal survival is improved by glia after 2 h but not 6 h OGD

We utilized oxygen–glucose deprivation (OGD), as described previously (Lin et al., 2016), to model ischemic stroke *in vitro*. To understand how various brain cell types interact after ischemic-like injury to potentially promote cell survival, we first determined the optimal length of OGD. Survival of neurons, astrocytes, and microglia grown separately was assessed after an extended period of ischemic-like injury (6 h OGD). We found that 24 h after the OGD challenge, neurons (Supplementary Figures 1a,d,e) and microglia (Supplementary Figures 1c–e) exhibited significant cell death after 6 h OGD by live/dead staining and decreased cell viability by MTT assay compared to normoxic controls. Astrocyte survival, however, was unchanged after 6 h OGD by the same methods (Supplementary Figures 1b,d,e), suggesting that astrocytes are less susceptible to harsh ischemic-like injury.

We next returned glia to the dish with neurons to more closely model physiological relevance. We utilized a transwell co-culture system to allow the analysis of neuronal viability without glial contamination (Supplementary Figure 1f). Neurons were plated on a dish alone (Supplementary Figure 1g), with astrocytes (Supplementary Figure 1h), microglia (Supplementary Figure 1i), or both astrocytes and microglia (Supplementary Figure 1j) on the transwell insert. After 6 h OGD, neuronal survival was significantly decreased compared to normoxic controls and remained unimproved in any condition (Supplementary Figures 1k,l), suggesting that this length of ischemic-like injury induced irreversible neuronal injury and death.

**Figure 1:**
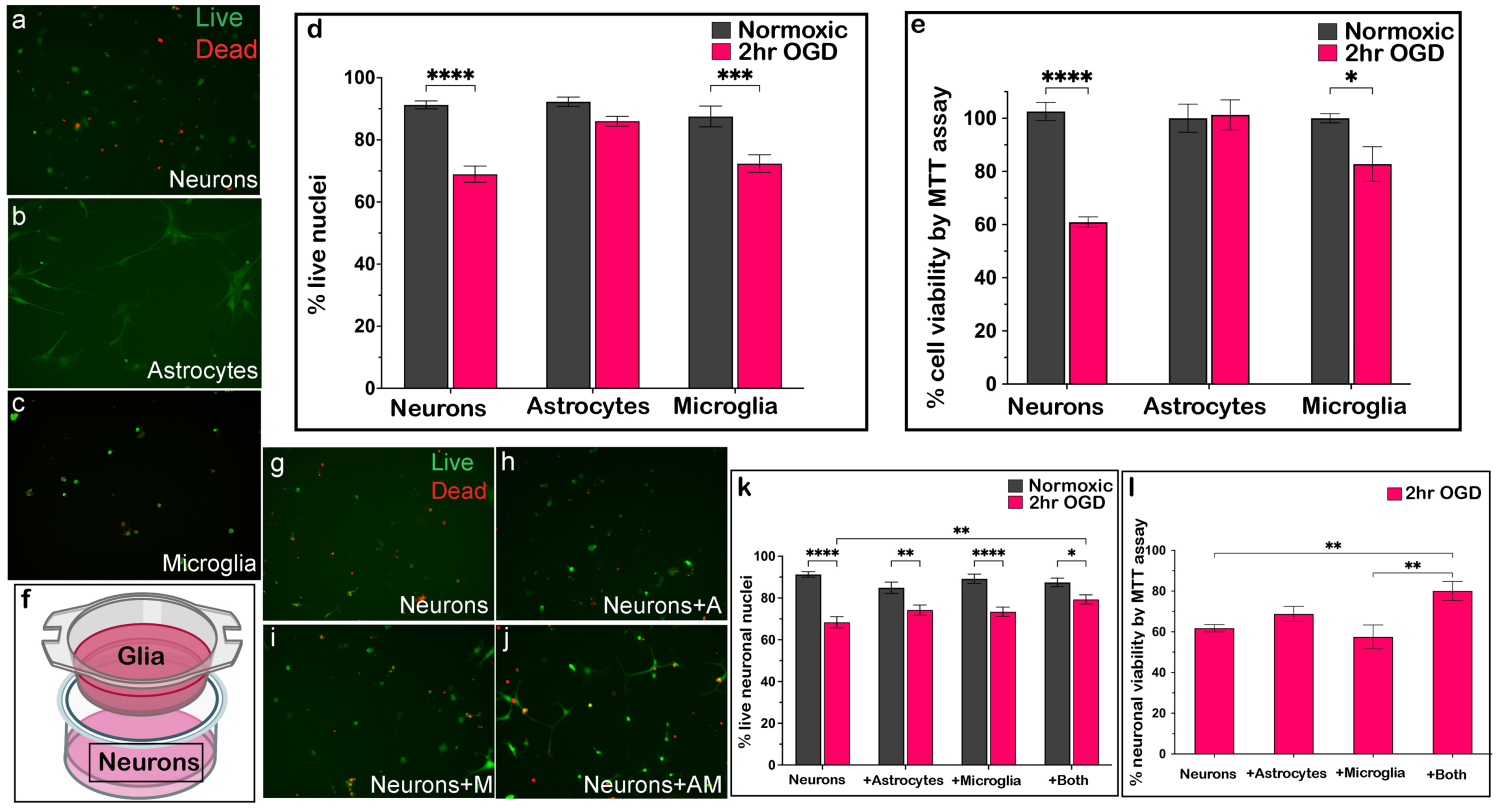
Two hours OGD induces cellular death *in vitro* that can be rescued by glia. After 2 h OGD (*n* = 6 biological replicates/cell type), there were significantly more live cells compared to 6 h OGD (Supplementary Figure 1) as measured by live/dead staining (20×; green = live cells; red = dead cells) **(a–d)** and MTT assay **(e)**. Neurons **(a,d)** and microglia **(c,d)** had significantly fewer live cells after OGD compared to normoxic, while astrocytes **(b,d)** did not. Changes in neuronal viability were confirmed by MTT assay (**e**; *n* = 6 biological replicates/cell type). Neuronal survival **(g)** after OGD was significantly improved with the addition of both astrocytes and microglia on a transwell **(f)** by live/dead staining (**j,k**; *n* = 24/condition) and MTT assay (**l**; *n* = 6 biological replicates/condition). There was no change in the number of live cells or neuronal viability with the addition of astrocytes **(h,k,l)** or microglia **(i,k,l)** alone. Analyzed by Student’s *t*-test, one-way ANOVA, or two-way ANOVA as appropriate. *p ≤ 0.05, **p ≤ 0.01, ***p ≤ 0.005, ****p ≤ 0.0001. Created in BioRender.

We next determined whether a more moderate ischemic-like injury (2 h OGD) provided an environment more conducive to neuronal rescue from damage/death. While there remained significant loss of live neurons (Figures 1a,d) and microglia (Figures 1c,d) by live/dead staining and a decline in cell viability by MTT assay (Figure 1e) compared to normoxic controls, survival of both cell types was greater after 2 h than 6 h OGD. Not surprisingly, 2 h OGD did not produce cell death (Figures 1b,d) or decreased cell viability (Figure 1e) in astrocytes, as seen with 6 h OGD.

When neuronal survival was assessed after 2 h OGD in co-cultures consisting of neurons in a dish with glia on transwell inserts (Figures 1f,g), we found neurons plated with both astrocytes and microglia contained significantly more live neurons (Figures 1j,k) as well as significantly improved neuronal viability (Figure 1l) compared to neurons grown with astrocytes (Figures 1h,k,l) or microglia only (Figures 1i,k,l). While neuronal viability did not improve to the level of normoxic controls (Figure 1k), our results nonetheless suggested that these three brain cell types can interact to promote neuronal survival *in vitro* after moderate ischemic-like injury.

### 3.2 Astrocyte P2Y1R KO promotes neuronal survival after OGD

**Figure 2:**
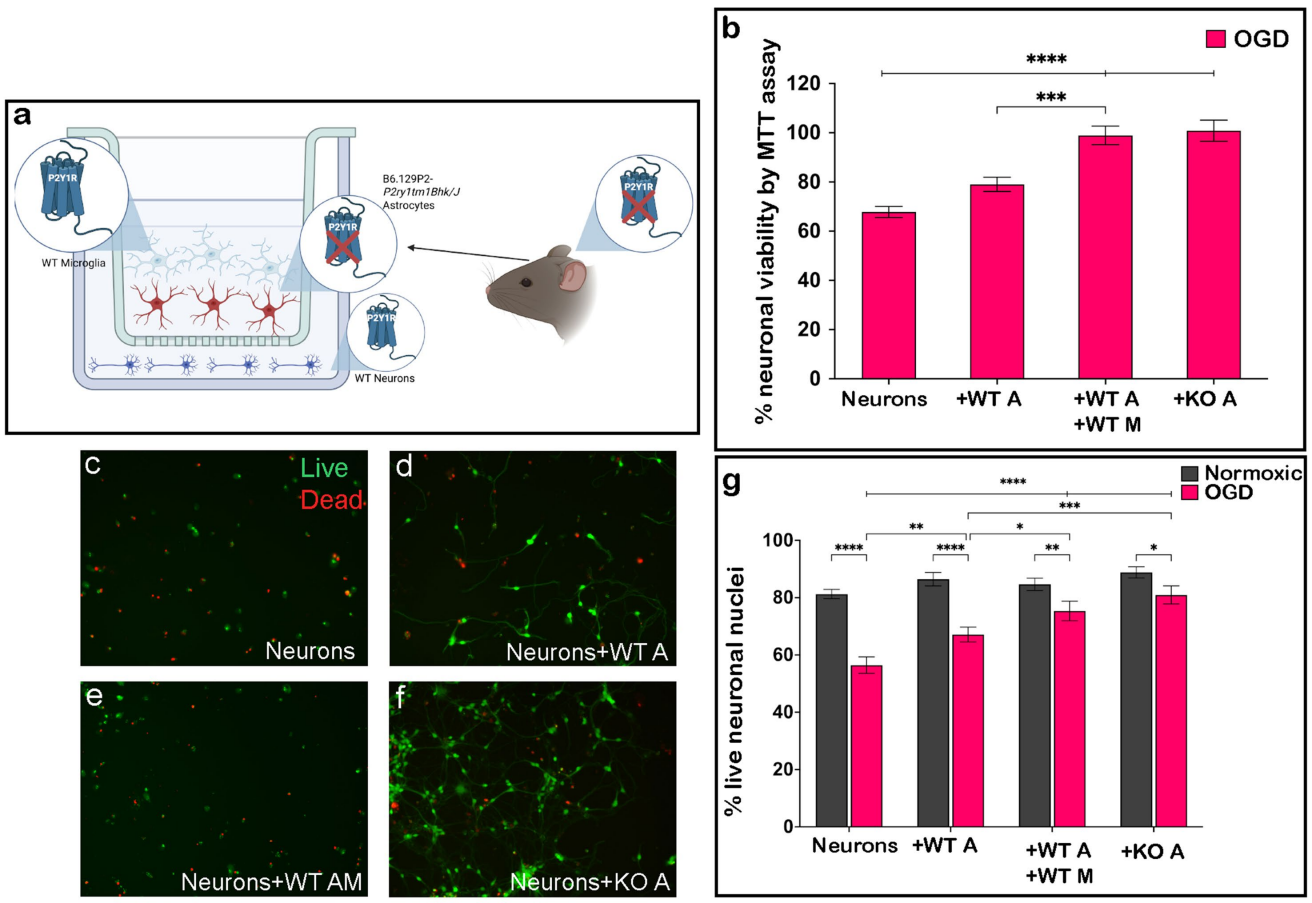
P2Y1R knockout astrocytes improve neuronal survival after OGD regardless of microglia. P2Y1R knockout astrocytes were cultured with neurons and microglia generated from wildtype animals in heterotypic cultures **(a)**. Knockout astrocytes improved neuronal viability regardless of microglial presence in the dish by MTT assay (**b**; *n* = 4 biological replicates/condition) and increased the number of live neuronal nuclei by live/dead staining (20×; **c–g**; *n* = 4 biological replicates/condition). The increase in neuronal survival with the addition of knockout astrocytes **(b,f,g)** was comparable to the increased viability **(b)** and live neuronal nuclei **(e,g)** seen in homotypic cultures of wildtype astrocytes and microglia and improved over cultures containing only wildtype astrocytes **(b,d,g)**. Analyzed by one-way ANOVA or two-way ANOVA. *p ≤ 0.05, **p ≤ 0.01, ***p ≤ 0.005, ****p ≤ 0.0001. Created in BioRender.

We next tested whether the enhanced neuronal survival seen after 2 h OGD in cultures containing neurons and glia was associated with changes in astrocyte P2Y1R, which was previously found to be neuroprotective in a TBI model (Shinozaki et al., 2017). We utilized heterotypic co-cultures of P2Y1R KO mouse astrocytes with WT neurons and microglia (depicted in Figure 2a) and assessed neuronal survival after 2 h OGD. Utilizing MTT assay (Figure 2b) and live/dead staining (Figures 2c–g), we found that even in the absence of microglia (Figures 2b,f,g), P2Y1R KO astrocytes significantly improved neuronal survival after 2 h OGD. In contrast, WT astrocytes alone (Figures 2b,d,g) did not affect neuronal viability, instead requiring the simultaneous presence of microglia in the dish for substantial enhancement in neuronal survival (Figures 2b,e,g).

### 3.3 Astrocyte P2Y1R decreases after OGD only when both neurons and microglia are present

**Figure 3:**
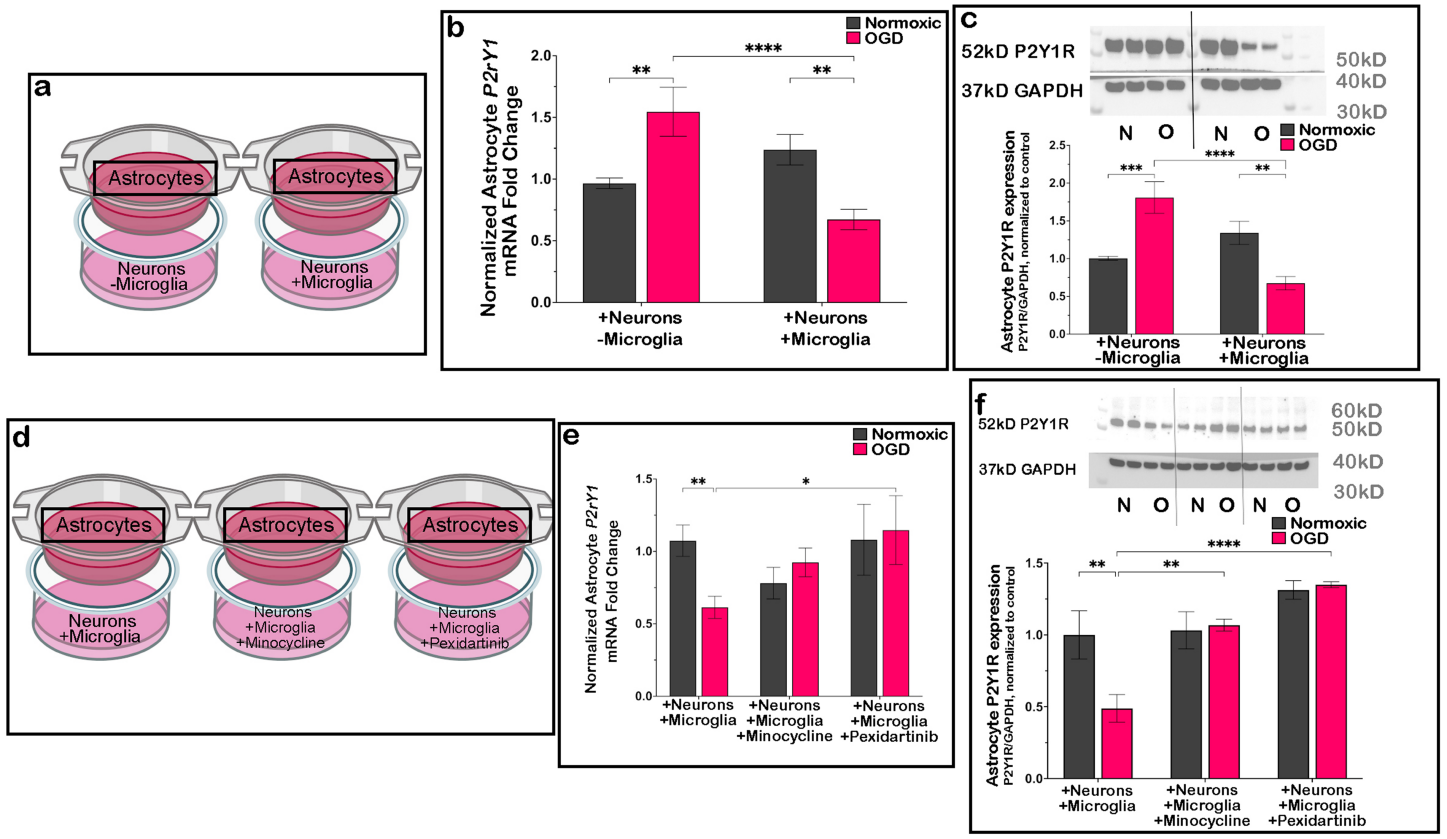
Astrocyte P2Y1R decreases after OGD only when microglia are present in culture. Following OGD, astrocytes co-cultured with neurons (**a**; boxed region indicates analyzed cell type) exhibited increased *P2ry1* mRNA (**b**; *n* = 5 biological replicates/condition) and P2Y1R protein expression (**c**; *n* = 5 biological replicates/condition) relative to normoxic controls. In contrast, when microglia were present in astrocyte/neuron co-cultures **(a)**, OGD resulted in a significant decrease in astrocyte *P2ry1* mRNA (**b**; *n* = 5 biological replicates/condition) and P2Y1R protein (**c**; *n* = 5 biological replicates/condition) compared with both normoxic controls and OGD cultures lacking microglia. Pharmacological depletion of microglia with Pexidartinib (CSF1R inhibitor; **d**; boxed region indicates analyzed cell type) prevented this effect, as astrocyte *P2ry1* mRNA (**e**; *n* = 4 biological replicates/condition) and P2Y1R protein levels (**f**; *n* = 4 biological replicates/condition) remained comparable to normoxic controls. Similarly, inhibition of microglial activation with minocycline (**d**; boxed region indicates analyzed cell type) preserved astrocyte *P2ry1* mRNA (**e**; *n* = 4 biological replicates/condition) and P2Y1R protein expression (**f**; *n* = 4 biological replicates/condition), preventing the OGD-induced downregulation observed in the presence of microglia. All data are presented with their expression relative to the normoxic condition without microglia or treatment. Analyzed by two-way ANOVA. *p ≤ 0.05, **p ≤ 0.01, ***p ≤ 0.005, ****p ≤ 0.0001. Created in BioRender.

As heterotypic co-cultures of P2Y1R KO astrocytes and WT neurons improved neuronal survival after OGD to the same extent as homotypic co-cultures containing WT astrocytes + microglia, we next investigated if the latter was also due to changes in astrocyte P2Y1R expression. To do so, we co-cultured neurons with astrocytes ± microglia from WT mice, plating neurons and microglia in the dish and astrocytes on a transwell insert (Figure 3a). After OGD, *P2ry1* RNA (Figure 3b) and P2Y1R protein (Figure 3c) expression increased in astrocytes co-cultured with neurons but without microglia as compared to normoxic controls. However, when microglia were also present in the dish, astrocyte *P2ry1* RNA and P2Y1R expression significantly decreased compared to both normoxic controls and astrocytes that underwent OGD without microglia (Figures 3b,c). This suggested a critical interaction between these cell types, with microglia inducing the downregulation of astrocyte P2Y1R only after injury.

As astrocyte P2Y1R expression decreased only when microglia were in the dish with astrocytes and neurons, we inhibited microglial function using two different inhibitors to confirm microglial importance in this mechanism (Figure 3d). First, samples were treated with minocycline, a tetracycline antibiotic, to prevent microglial activation (Seabrook et al., 2006). With minocycline treatment, *P2ry1* RNA (Figure 3e) and P2Y1R (Figure 3f) protein expression were unchanged after OGD compared to normoxic conditions. Importantly, minocycline treatment prevented the downregulation of *P2ry1* RNA (Figure 3e) and P2Y1R protein expression (Figure 3f) previously seen after OGD when microglia were present (Figures 3a–c). Next, samples were treated with Pexidartinib, a CSF1R inhibitor that depletes microglia since microglial maintenance is CSF1R-dependent (Okojie et al., 2023). Like the effect observed with minocycline, Pexidartinib prevented the downregulation of *P2ry1* RNA (Figure 3e) and P2Y1R protein expression (Figure 3f). Consistent with previous results, this suggests microglial activation is necessary for the downregulation of astrocyte P2Y1R during ischemic-like injury.

### 3.4 Neuronally-released ATP induces changes in astrocyte P2Y1R through microglial activation

**Figure 4:**
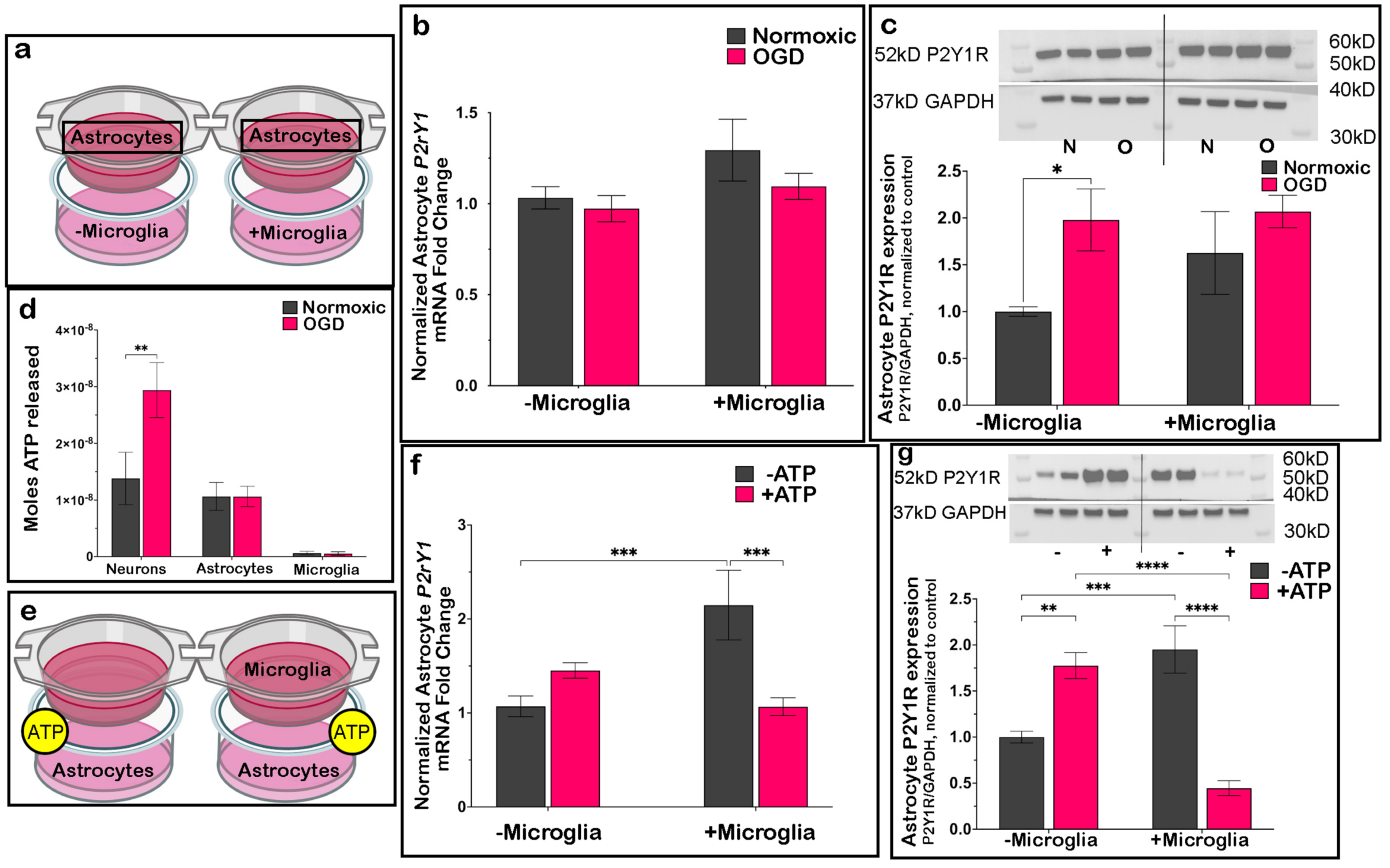
Dying neurons release ATP to induce downregulation of astrocyte P2Y1R expression. When neurons were removed from the dish (**a**; box indicates cell type being analyzed), astrocyte *P2ry1* RNA (**b**; *n* = 5 biological replicates/condition) and P2Y1R protein expression (**c**; *n* = 4/condition) did not change after OGD regardless of microglial presence. Neurons, but not astrocytes or microglia, release significant amounts of ATP extracellularly after OGD as measured by a luciferase assay (**d**; *n* = 4 biological replicates/condition). Exogenous ATP (10^−10^ mol) added to co-cultures of astrocytes and microglia for 24 h in place of OGD **(e)** resulted in a downregulation of *P2ry1* RNA (**f**, *n* = 4 biological replicates/condition) and P2Y1R protein expression (**g**; *n* = 4 biological replicates/condition) compared to normoxic control as well as astrocytes alone treated with ATP. All expression data is presented relative to the normoxic condition without microglia or no ATP condition – microglia. Analyzed by Student’s *t*-test, one-way ANOVA, or two-way ANOVA as appropriate; *p ≤ 0.05, **p ≤ 0.01, ***p ≤ 0.005, ****p ≤ 0.0001. Created in BioRender.

To test if neurons also played a critical role in the observed changes, neurons were eliminated from the co-cultures. Importantly, after OGD with astrocytes in a well and microglia on the transwell (Figure 4a), astrocyte *P2ry1* RNA (Figure 4b) or P2Y1R protein expression (Figure 4c) did not decrease if neurons were absent from glial co-cultures. This suggested that in ischemic-like injury, dying neurons may be necessary to activate microglia to produce the downregulation of astrocyte P2Y1R.

As it is known that dying cells release ATP (Burnstock, 2016; Fields and Burnstock, 2006), we tested if ATP could modify microglial phenotype to reduce astrocyte P2Y1R. Using a luciferase assay, we measured ATP concentration released from each cell type in our co-cultures after OGD. Neurons, but not astrocytes or microglia, released significantly more ATP compared to normoxic controls (Figure 4d), consistent with its potential role as the trigger for the mechanism to reduce astrocyte P2Y1R. We then plated and treated cells with a dose of ATP comparable to what neurons released after OGD (3 × 10^−8^ mols ATP). As seen with OGD (Figures 4b,c), astrocytes alone treated with ATP (Figure 4e) increased *P2ry1* RNA (Figure 4f) and P2Y1R (Figure 4g) protein expression compared to normoxic controls. Interestingly, a similar increase in *P2ry1* RNA (Figure 4f) and P2Y1R (Figure 4g) protein expression was observed under normoxic conditions when microglia were added to astrocyte cultures. However, after ATP treatment of astrocyte: microglial co-cultures, there was a significant decrease in *P2ry1* RNA (Figure 4f) and P2Y1R (Figure 4g) protein expression compared to both normoxic controls and astrocytes alone treated with ATP. These findings, which were comparable to those found with OGD (Figures 4a–c), support the premise that ATP released from dying ischemic neurons initiates the microglial mechanism that leads to a reduction in astrocyte P2Y1R.

### 3.5 B-NGF and IL-2 released by microglia induce downregulation of astrocyte P2Y1R

As neurons and microglia were necessary to see the downregulation of astrocyte P2Y1R, we next investigated potential cytokines and growth factors released from these cells (Supplementary Figure 2a) that might mediate these changes. Using a cytokine array, we found that after 2 h OGD, 16 of the 32 targets tested were upregulated (Supplementary Figure 2b). Each target was then exogenously added to astrocyte cultures to determine if any could mimic microglial modulation of astrocyte P2Y1R expression. Using Western blotting (Supplementary Figure 2c), we found an increase in astrocyte P2Y1R protein expression in samples treated with exogenous Activin A (Supplementary Figure 2d), Agrin (Supplementary Figure 2e), B7-2/CD86 (Supplementary Figure 2f), ICAM-1 (Supplementary Figure 2g), IL-10 (Supplementary Figure 2h), or Fas ligand (Supplementary Figure 2i). Additionally, we found no change in astrocyte P2Y1R protein expression in samples treated with CINC-1 (Supplementary Figure 3b), Fractalkine (Supplementary Figure 3c), IFN-g (Supplementary Figure 3d), IL-1a (Supplementary Figure 3e), IL-1b (Supplementary Figure 3f), IL-6 (Supplementary Figure 3g), TNF-a (Supplementary Figure 3h), or VEGF (Supplementary Figure 3i).

**Figure 5:**
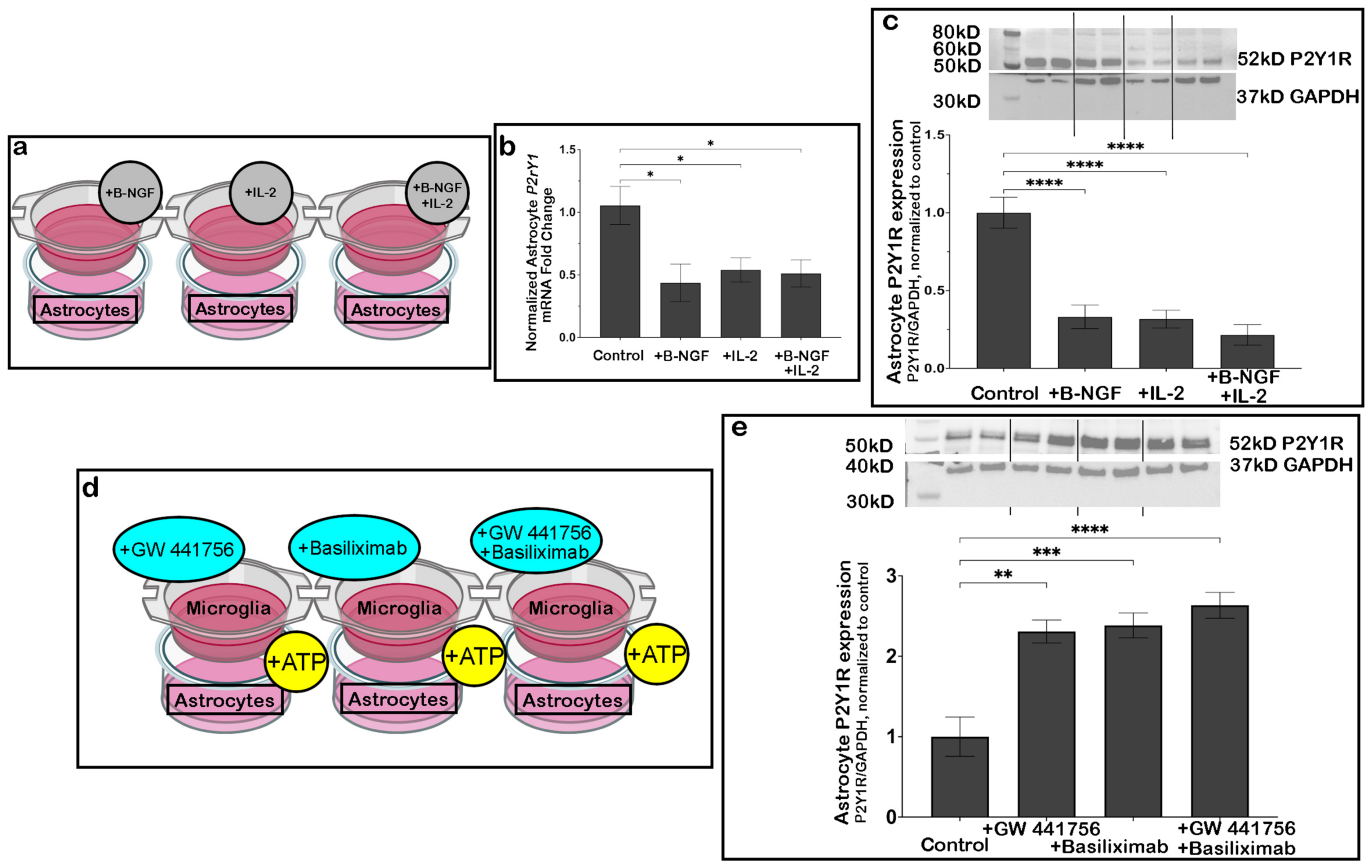
Exogenous B-NGF or IL-2 treatment decreased astrocyte P2Y1R expression. Astrocytes were separately treated with exogenous B-NGF, IL-2, or B-NGF and IL-2 together **(a)**. Samples treated with B-NGF alone, IL-2 alone, or B-NGF and IL-2 together decreased *P2ry1* RNA expression by qPCR (**b**; *n* = 4 biological replicates/condition) and decreased P2Y1R protein expression by western blotting, relative to untreated astrocytes (**c**; *n* = 3 biological replicates/condition). During ATP treatment, astrocytes and microglia were treated with inhibitors of B-NGF (GW 441756) and IL-2 (Basiliximab) both separately and in conjunction with one another to prevent signaling (**d**; *n* = 3 biological replicates/condition; box indicates cell type being analyzed). All treatment conditions increased P2Y1R expression as assessed by western blotting (**e**; *n* = 3 biological replicates/condition); analyzed by one-way ANOVA; *p < 0.05, **p < 0.01, ***p ≤ 0.001, ****p ≤ 0.0001. Created in BioRender.

However, the addition of 2 factors, B-NGF or IL-2, to astrocytes (Figure 5a) mimicked injury-induced downregulation of astrocyte *P2ry1* RNA (Figure 5b) and P2Y1R protein (Figure 5c), replicating the effects of microglia in our earlier OGD co-culture studies. Interestingly, there was not an additive or synergistic downregulation when both B-NGF and IL-2 were added to astrocytes together (Figures 5b,c). This suggests that the two factors may converge on the same downstream pathway to induce the reduction in astrocyte P2Y1R. As we previously found that neuronally-released ATP is important in triggering this mechanism after OGD, we then inhibited the B-NGF receptor, TrkA, with the inhibitor GW 441756 (2.0 nM, according to manufacturer’s recommendation) and the IL-2 receptor, IL-2R, with Basilimiximab (3.5 nM, according to manufacturer’s recommendation) either separately or together and repeated the experiment utilizing exogenous ATP (Figure 5d). In these cultures, inhibiting TrkA or IL-2R separately prevented the downregulation of astrocyte P2Y1R (Figure 5e). However, simultaneous inhibition of TrkA and IL-2R did not further enhance the downregulation of P2Y1R, supporting the likelihood that TrkA and IL-2R signaling share a downstream pathway.

### 3.6 TrkA expression rises transiently in astrocytes after OGD

**Figure 6:**
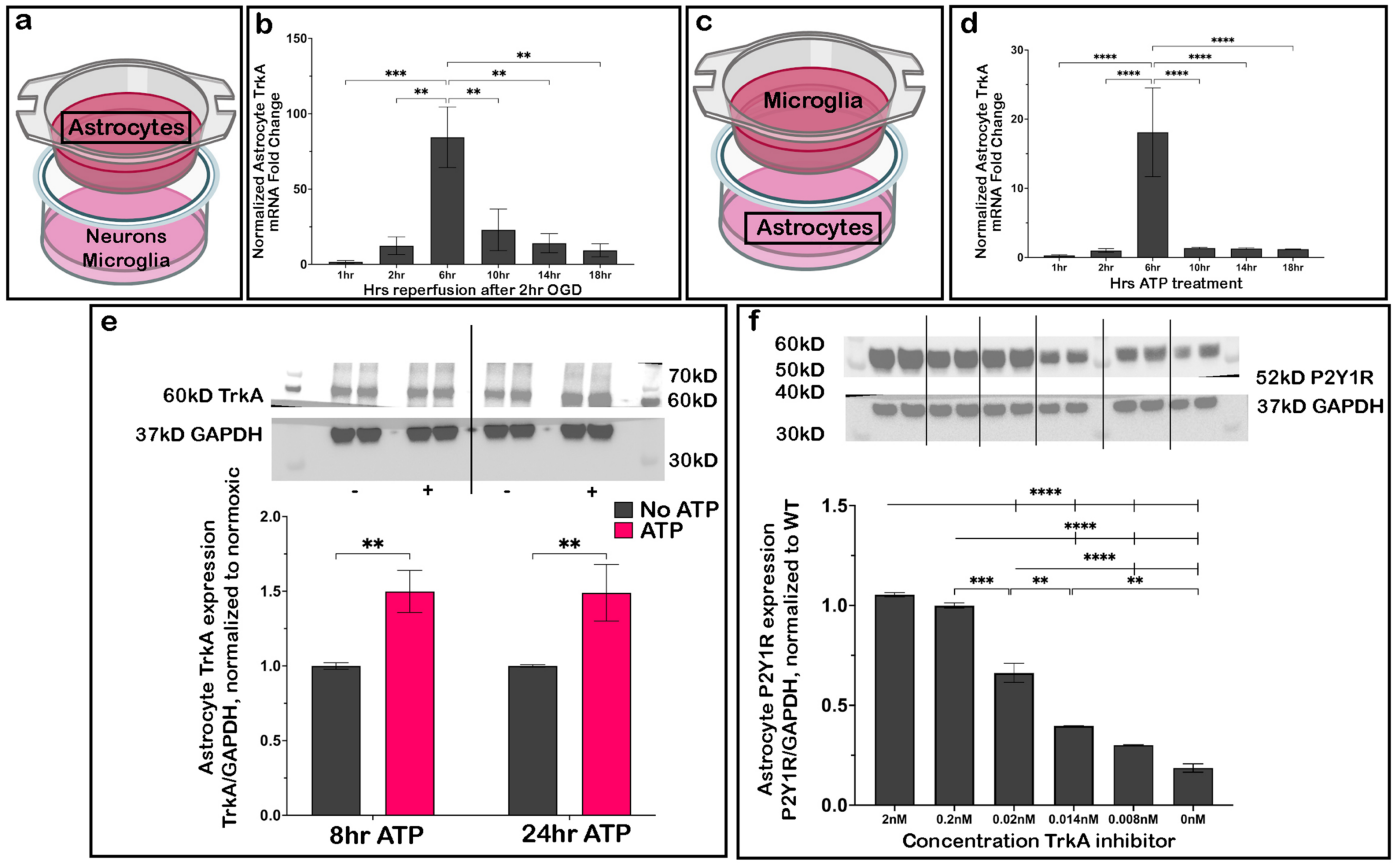
P2Y1R expression decreases dose-dependently with a TrkA inhibitor after 24 h ATP treatment. Astrocytes co-cultured with neurons and microglia **(a)** showed an increase in *TrkA* RNA expression 6 h after OGD (**b**; *n* = 3 biological replicates/condition). Similarly, after 6 h ATP treatment astrocytes co-cultured with microglia **(c)** had increased *TrkA* RNA expression (**d**; *n* = 3 biological replicates/condition). As OGD and exogenous ATP treatment had a similar effect, ATP was used in later experiments. Exogenous ATP (10^^-10^ mol) added to co-cultures of astrocytes and microglia for 8 and 24 h (**e**; *n* = 3 biological replicates/condition) resulted in an increase in TrkA protein expression. After high dose treatment of the TrkA inhibitor GW 441756 (2 nM and 0.2 nM) during 24 h ATP treatment, astrocyte P2Y1R expression was unchanged (**f**; *n* = 3 biological replicates/condition). As the dosage of TrkA inhibitor decreased, however, astrocyte P2Y1R decreased in a dose-dependent manner. Analyzed by one-way ANOVA or Student’s *t*-test as appropriate; *p ≤ 0.05, **p ≤ 0.01, ***p ≤ 0.005, ****p ≤ 0.0001. Created in BioRender.

While our results indicated that B-NGF and IL-2 released by microglia are two possible mediators initiating P2Y1R downregulation in astrocytes, we focused on how B-NGF signaling induced astrocyte P2Y1R downregulation. As such, we next examined expression of the B-NGF receptor TrkA in astrocytes. To do so, neurons and microglia were plated in the dish with astrocytes on a transwell (Figure 6a) and subjected to OGD. We then assessed astrocyte *TrkA* RNA expression throughout the reperfusion period. We observed a dramatic rise in astrocyte *TrkA* expression after 6 h reperfusion that was diminished at later times (Figure 6b). We then used exogenous ATP treatment in place of OGD as done previously (Figure 6c). By mimicking neuronally released ATP in the absence of neurons, the role of neuronal TrkA as a confounding variable was eliminated. Similar to the OGD condition, there was a significant increase in astrocyte *TrkA* RNA expression after 6 h ATP treatment (Figure 6d) and a concomitant rise in TrkA protein expression several hours later (8 h) (Figure 6e). In contrast to the transient spike observed in astrocyte *TrkA* mRNA, TrkA protein remained elevated even after 24 h ATP treatment (Figure 6e).

Based on this finding, we then utilized a specific TrkA inhibitor, GW 441756, with an IC_50_ of 0.2 nM to block the NGF receptors (Wood et al., 2004). To test whether blockade of NGF receptors prevented the downregulation of astrocyte P2Y1R, we plated astrocytes and microglia with varying concentrations of the inhibitor throughout 24 h ATP treatment. We found that with an inhibitor concentration at 0.2 nM or greater 2.0 nM, the expected decrease in P2Y1R protein expression was blocked (Figure 6f). However, at concentrations below 0.2 nM, a dose-dependent decrease in astrocyte P2Y1R expression was observed as the potency of inhibitor declined. Taken together, these findings are consistent with a possible role for B-NGF and its receptor in the downregulation of astrocyte P2Y1R (Figure 6f).

### 3.7 TrkA^F592A^ astrocytes do not protect neurons after ischemic-like injury

**Figure 7:**
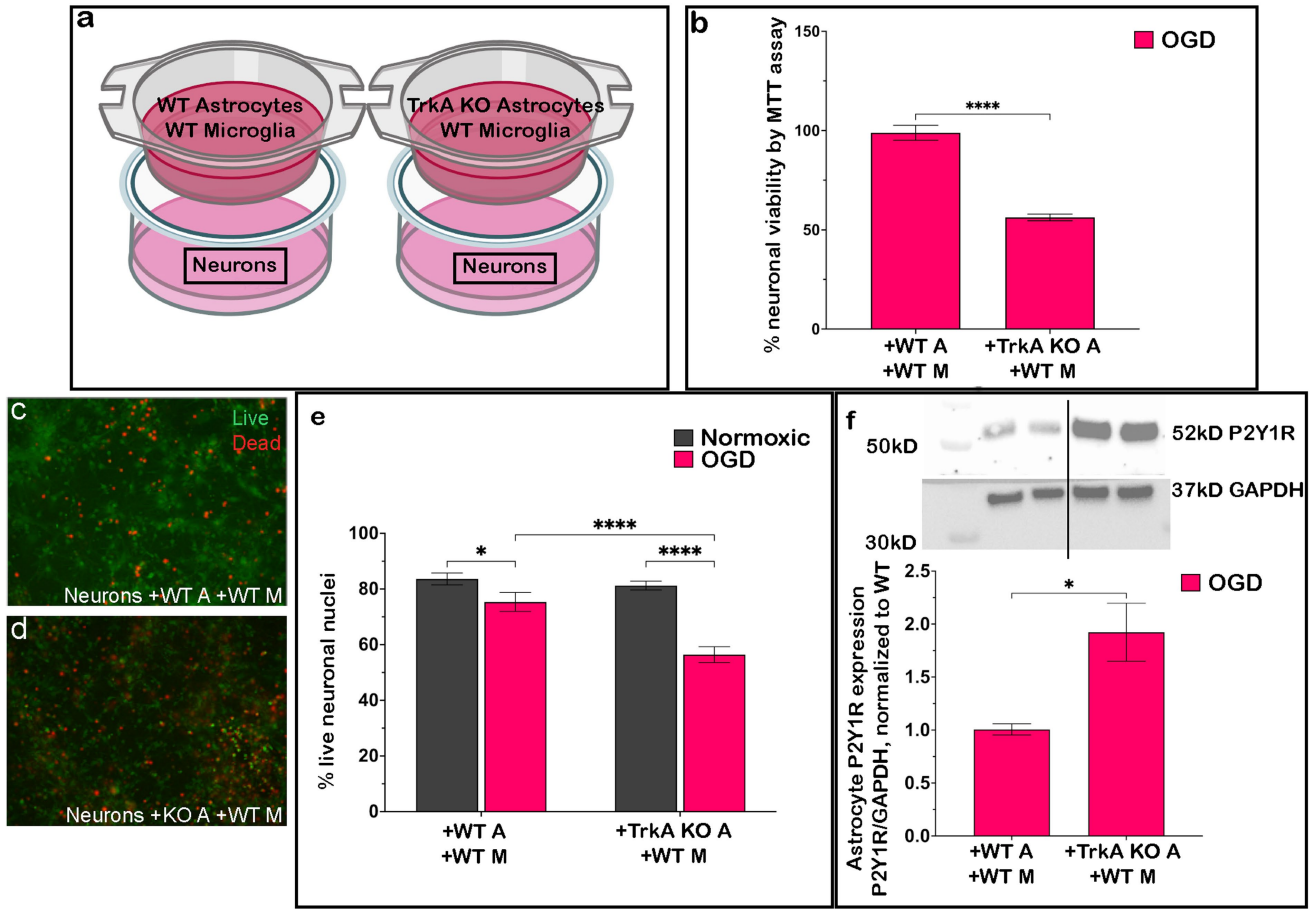
TrkA^F592A^ astrocytes do not protect neurons from OGD. Neurons co-cultured with TrkA^F592A^ astrocytes and WT microglia **(a)** have decreased neuronal viability after OGD by MTT assay (**b**; *n* = 3 biological replicates/condition) and live/dead staining (20x; **d,e**; *n* = 3 biological replicates/condition) compared to neurons cultured with WT astrocytes and WT microglia **(b,c,e)**. TrkA^F592A^ astrocytes have higher P2Y1R expression than WT astrocytes after 24 h ATP treatment (**f**; *n* = 3 biological replicates/condition). Analyzed by Student’s *t*-test or 2-way ANOVA as appropriate; *p ≤ 0.05, ****p ≤ 0.0001. Created in BioRender.

As pharmacological inhibition prevented astrocyte P2Y1R downregulation after injury, we utilized astrocytes cultured from TrkA^F592A^ mice treated with 1NMPP1 to block TrkA signaling specifically in these astrocytes and assessed if the cells were neuroprotective after OGD. Neurons were plated with WT microglia and either WT astrocytes or TrkA^F592A^ astrocytes (Figure 7a) and viability was measured by MTT assay (Figure 7b) or live/dead staining (Figures 7c–e). Neuronal survival was decreased when plated when TrkA^F592A^ astrocytes compared to WT astrocytes through both methods. Additionally, when treated with ATP, there was an increase in astrocyte P2Y1R in TrkA^F592A^ astrocytes relative to WT astrocytes (Figure 7f). Taken together, this supports the notion that astrocyte TrkA is vital for the neuroprotective mechanism.

### 3.8 Astrocytes + microglia or P2Y1R KO astrocytes decreases intracellular calcium levels in neurons after OGD

As P2Y1R activity is known to induce changes in intracellular calcium (Belov Kirdajova et al., 2020; Del Puerto et al., 2013), we utilized heterotypic co-cultures of P2Y1R KO mouse astrocytes with WT neurons or homotypic cultures of WT astrocytes ± microglia and measured neuronal intracellular calcium after 2 h OGD followed by 24 h reperfusion (Supplementary Figure 4a). Neurons cultured with WT astrocytes + microglia had significantly lower intracellular concentrations than neurons cultured with WT astrocytes alone (Supplementary Figure 4b). Importantly, this reduction in neuronal intracellular calcium was mimicked in neurons cultured only with P2Y1R KO astrocytes (Supplementary Figure 4b).

To test whether intracellular Ca^2+^ mediates neuronal death, we pharmacologically manipulated calcium dynamics during OGD. Buffering intracellular Ca^2+^ with BAPTA significantly increased neuronal survival, as measured by both live/dead staining (Supplementary Figures 4d,f) and MTT assay (Supplementary Figure 4g), compared to OGD alone (Supplementary Figures 4c,f,g). In contrast, blockade of NMDA receptors with dizocilpine maleate (Supplementary Figures 4e,f,g) did not improve neuronal viability, suggesting that NMDA receptor–mediated Ca^2+^ influx is not the primary driver of cell death in this model.

Together, these data demonstrate that intracellular Ca^2+^ accumulation is involved in OGD-induced neuronal death. Moreover, the reduction in neuronal Ca^2+^ observed with astrocytic P2Y1R knockout is consistent with a mechanism in which astrocytic signaling regulates neuronal survival by modulating Ca^2+^ homeostasis through a pathway that is independent of NMDA receptor activity.

## 4 Discussion

Overall, the results of the present study demonstrate that damaged ischemic neurons released high levels of ATP to activate microglia after OGD. Importantly, these activated microglia secreted higher levels of NGF and IL-2 than normoxic controls, signaling downstream TrkA and IL2 receptors, resulting in a consequent downregulation in astrocyte P2Y1R. These critical interactions were further accompanied by a decline in intracellular calcium levels in neurons and enhanced neuronal survival after OGD (see Supplementary Figure 5). Importantly, the rescue of neurons from OGD was mimicked by P2Y1R knockout astrocytes in culture. In this work, we demonstrate that the molecular crosstalk between neurons, astrocytes, and microglia is necessary to produce the downregulation in astrocyte P2Y1R expression, thereby reducing neuronal calcium burden which underlies the rescue of neurons in *in vitro* models of stroke.

In exploring the individual elements at play in this process, we utilized a transwell system *in vitro,* which allowed us to separate and recombine cell types in various ways to probe their individual role in neuronal plasticity. Additionally, it granted us the ability to isolate specific cells of interest for subsequent analysis. In so doing, we found that damage to neurons and release of ATP is an essential trigger to the neuroprotective mechanism, a finding supported by earlier studies on stroke (Carmo et al., 2014; Chin et al., 2013; Fukumoto et al., 2019; Kijima et al., 2024; Lammer et al., 2011; Sun et al., 2008; Zheng et al., 2013) and TBI (Fujita et al., 2009; Liston et al., 2020; Moro et al., 2021; Neary et al., 2003; Shinozaki et al., 2005; Talley Watts et al., 2013). As such, in our experiments, high concentrations of exogenous ATP were able to substitute for damaged neurons in the system. We further showed in our transwell cultures that ATP acts directly on astrocytes to increase P2Y1R, regardless of the presence of microglia, thus negatively affecting stroke recovery and further reinforcing a critical role for ATP in this mechanism.

In addition to the importance of ATP, we determined that microglia were also vital to the downregulation of astrocyte P2Y1R. We found that when microglia were present with astrocytes, astrocyte P2Y1R decreased. Further supporting the significance of microglia, treatment with either minocycline to prevent microglial activation or Pexidartinib to deplete microglia prevented this decrease. Moreover, neuronal survival increased only when astrocytes and microglia were both present in our transwell system or when P2Y1R knockout astrocytes were present, thereby eliminating the necessity for microglia. Thus, microglia play a critical role in reversing the adverse effects of high astrocyte P2Y1R caused by ATP from damaged neurons. This contrasts with a previous study that found P2Y1R blockade had a direct neuroprotective effect on neurons (Carmo et al., 2014). The results of our study instead suggest that P2Y1R-mediated recovery occurs through a multi-step, multi-cell mechanism and implicates a critical non-cell autonomous role for astrocyte P2Y1R in neuroprotection.

To understand how microglia induce the downregulation of astrocyte P2Y1R, we investigated potential mediators of this cellular interaction. P2Y1R signaling has previously been implicated in glial scar formation after stroke and TBI (Kijima et al., 2024; Shinozaki et al., 2017). Our work found that pro-inflammatory molecules (i.e., TNF-a) associated with glial scarring upregulated astrocyte P2Y1R. In contrast, we found that either the growth factor NGF or the cytokine IL-2 released from activated microglia was able to prevent an upregulation of astrocyte P2Y1R after injury while their receptor antagonists (GW 441756, Basiliximab respectively) reversed this neuroprotective effect. Interestingly, no additive/synergistic effect was noted when astrocytes were treated simultaneously with NGF and IL-2, suggesting that these signaling pathways functionally converge.

While NGF and IL-2 are members of distinctive molecular classes with distinctive receptors, they possess similar intracellular effectors such as PI3K/Akt, MAPK/ERK, or transcriptional regulators such as CREB or NF-κB (Kaplan and Miller, 2000; Malek and Castro, 2010). Therefore, it is possible that TrkA and IL-2 signaling converge upstream of P2Y1R and regulate astrocytic Ca^2+^ signaling competence so that inhibition of either pathway may be sufficient to impair IP₃-dependent Ca^2+^ responses, thereby limiting downstream P2Y1R-mediated signaling and explaining the lack of additivity with combined inhibition (Agulhon et al., 2008; Bazargani and Attwell, 2016).

In this work, there was a concomitant increase in astrocyte TrkA after both OGD and ATP injury. However, there was a transient increase in *TrkA* mRNA at 6 h but sustained TrkA protein expression for at least 24 h. Temporal dissociation between transcript and protein dynamics has been reported previously in CNS injury models. In transient forebrain ischemia, *BDNF* mRNA shows early transient elevation while protein changes evolve on a different time course across regions, consistent with delayed or region-specific protein regulation (Kokaia et al., 1995; Kokaia et al., 1998). In seizure models, early increases in BDNF mRNA have been observed alongside more prolonged elevations in protein levels, supporting a pattern in which transcripts peak and decline before protein levels stabilize (Nawa et al., 1995). More generally, protein expression may be delayed or outlast changes in mRNA due to differences in translation and turnover rates (Vogel and Marcotte, 2012). Therefore, the sustained TrkA protein levels we observe after OGD/ATP may reflect regulated translation or receptor stability rather than continued high-level transcription.

While it is likely that microglial mediators differ depending on the varied functions of P2Y1R signaling in the injured brain, in the context of our study, it is reasonable that NGF, possessing both trophic and anti-inflammatory effects and causing a concomitant rise in astrocyte TrkA expression, would promote neuroprotection. Supporting this notion, microglial secreted pro-NGF and NGF have also been implicated in other models of injury and neurodegeneration (Garcez et al., 2017; Seabrook et al., 2006; Yune et al., 2007). While we focused primarily on NGF signaling within this work, it remains an important avenue to further study the role of IL-2 and this convergence in the future. Additionally, *in vivo* studies in a mouse model of stroke are necessary and underway to confirm the biological relevance of this mechanism and to parse the mechanistic roles of B-NGF and IL-2 in neuroprotection through the downregulation of astrocyte P2Y1R.

To determine whether reduced neuronal Ca^2+^ contributes mechanistically to the protective effect of astrocytic P2Y1R loss, we directly manipulated calcium dynamics during OGD. Importantly, the magnitude of neuroprotection achieved by intracellular Ca^2+^ buffering was comparable to that observed with astrocytic P2Y1R knockdown, supporting the conclusion that reduced neuronal Ca^2+^ is sufficient to phenocopy the protective effect. Together, these findings demonstrate that the decrease in neuronal Ca^2+^ is not merely a marker of reduced excitotoxicity, but a functional mediator of neuronal survival in this model.

In this study, we describe a non-cell autonomous mechanism of endogenous neuroprotection in ischemic injury. Damaged neurons release ATP that acts on astrocytes and microglia. This induces a detrimental upregulation in astrocyte P2Y1R. However, activated microglia prevent this upregulation through the release of NGF (and IL-2), which acts on astrocyte TrkA receptors. Additionally, there is a decrease in neuronal intracellular calcium when P2Y1R expression is lacking in astrocytes, suggesting this may contribute to the observed neuroprotection. By understanding these inherent non-cell autonomous mechanisms and their molecular mediators, it may be possible to improve intrinsic neuroprotection and recovery from stroke.

## Data Availability

The original contributions presented in the study are included in the article/Supplementary material, further inquiries can be directed to the corresponding author.

## Supplementary material

Supplementary Figure 1 6hr OGD induces major cellular death *in vitro* which is not rescued by glia. Neurons, astrocytes, and microglia (*n*=24/cell type) were plated separately to determine their survivability after ischemic-like injury. There were significantly fewer live cells as measured by live/dead staining (green = live cells; red = dead cells) in neuron **(a, d)** and microglia **(c, d)** cultures after compared to normoxic conditions. Astrocytes **(b, d)** did not exhibit significant cell death under either condition. These findings were also confirmed by MTT assay (**e**; *n*=24/cell type). Neuronal cell death after 6hr OGD was further assessed with and without the addition of glial cell types using transwells that allow cells to share media but not directly interact (**f**; box indicates cell type being analyzed). Neuronal survival declined regardless of additional glial type by live/dead staining (**g–k**; *n*=24/condition) and MTT assay (**l**; *n*=24/condition). Analyzed by Student’s t-test, one-way ANOVA, or two-way ANOVA as appropriate. ***p*<0.01; *****p*<0.0001.

Supplementary Figure 2 Both pro- and anti-inflammatory cytokines and growth factors are released from neuron and microglia co-cultures after 2hr OGD. Cytokines and growth factors released from co-cultures of neurons and microglia **(a)** after 2hr OGD were measured using a cytokine array (**b**; n=6/condition). Upregulated factors were then exogenously added to astrocyte samples and P2Y1R expression was assessed using Western blotting **(c)**. There was an increase in astrocyte P2Y1R expression in samples with exogenously added Activin A **(d)**, Agrin **(e)**, B7-2/CD86 **(f)**, ICAM-1 **(g)**, IL-10 **(h)**, or Fas ligand **(i)**. Analyzed by student’s t-test; ** *p*< 0.01; *** *p*< 0.005; **** *p*< 0.0001.

Supplementary Figure 3 Exogenous addition of cytokines and growth factors does not decrease astrocyte P2Y1R. Additional upregulated cytokines and growth factors were exogenously added to astrocyte samples and P2Y1R expression assessed using Western blotting **(a)**. There was no change in astrocyte P2Y1R expression in samples with exogenously added CINC-1 **(b)**, Fractalkine **(c)**, IFN-g **(d)**, IL-1a **(e)**, IL-1b **(f)**, IL-6 **(g)**, TNF-a **(h)**, or VEGF **(i)**. Analyzed by student’s t-test; ** *p*< 0.01; *** *p*< 0.005; **** *p*< 0.0001.

Supplementary Figure 4 Neurons plated with WT astrocytes have increased intracellular calcium concentrations compared to neurons plated with WT astrocytes and microglia or P2Y1R KO astrocytes. WT neurons were plated on a dish and using a transwell co-cultured with homotypic WT astrocytes + microglia, or heterotypic KO astrocytes **(a)**. After 2hr OGD and 24hr reperfusion, intracellular calcium concentration was measured. Neurons cultured only with KO astrocytes or with WT astrocytes and microglia had significantly lower levels of intracellular calcium compared to samples cultured with WT astrocytes (**b**; *n*=8/condition). Representative live/dead images (green = live cells; red = dead cells) of neuronal cultures following OGD under control conditions **(c)**, with intracellular Ca²^+^ buffering **(d)**, or with NMDA receptor blockade **(e)**. OGD resulted in a significant reduction in neuronal survival compared to normoxic conditions, as assessed by live/dead staining, which was significantly rescued by treatment with BAPTA-AM but not by treatment with Dizocilpine maleate (**f**; *n* = 8/condition). These findings were further confirmed by MTT assay (**g**; *n* = 12/condition), where intracellular Ca²^+^ buffering significantly improved neuronal viability following OGD, whereas NMDA receptor inhibition did not confer protection. Analyzed by one-way ANOVA; **p*< 0.05; ** *p*< 0.01; ****p* < 0.001; *****p* < 0.0001.

Supplementary Figure 5 Graphical abstract. Damaged ischemic neurons release high levels of ATP to activate microglia after OGD. These activated microglia secrete higher levels of NGF and IL-2, signaling downstream TrkA and IL-2 receptors, and resulting in a consequent downregulation in astrocyte P2Y1 receptors. These critical interactions were further accompanied by a decline in intracellular calcium levels in neurons and enhanced neuronal survival after OGD.
