# Supplementary figures and images for: Non-cell autonomous downregulation of the purinergic receptor P2Y1R promotes neuroprotection after ischemic injury
Source: Front Cell Neurosci. 2026 Apr 21;20:1790325. doi: 10.3389/fncel.2026.1790325 (PMC13138967; doi:10.3389/fncel.2026.1790325)

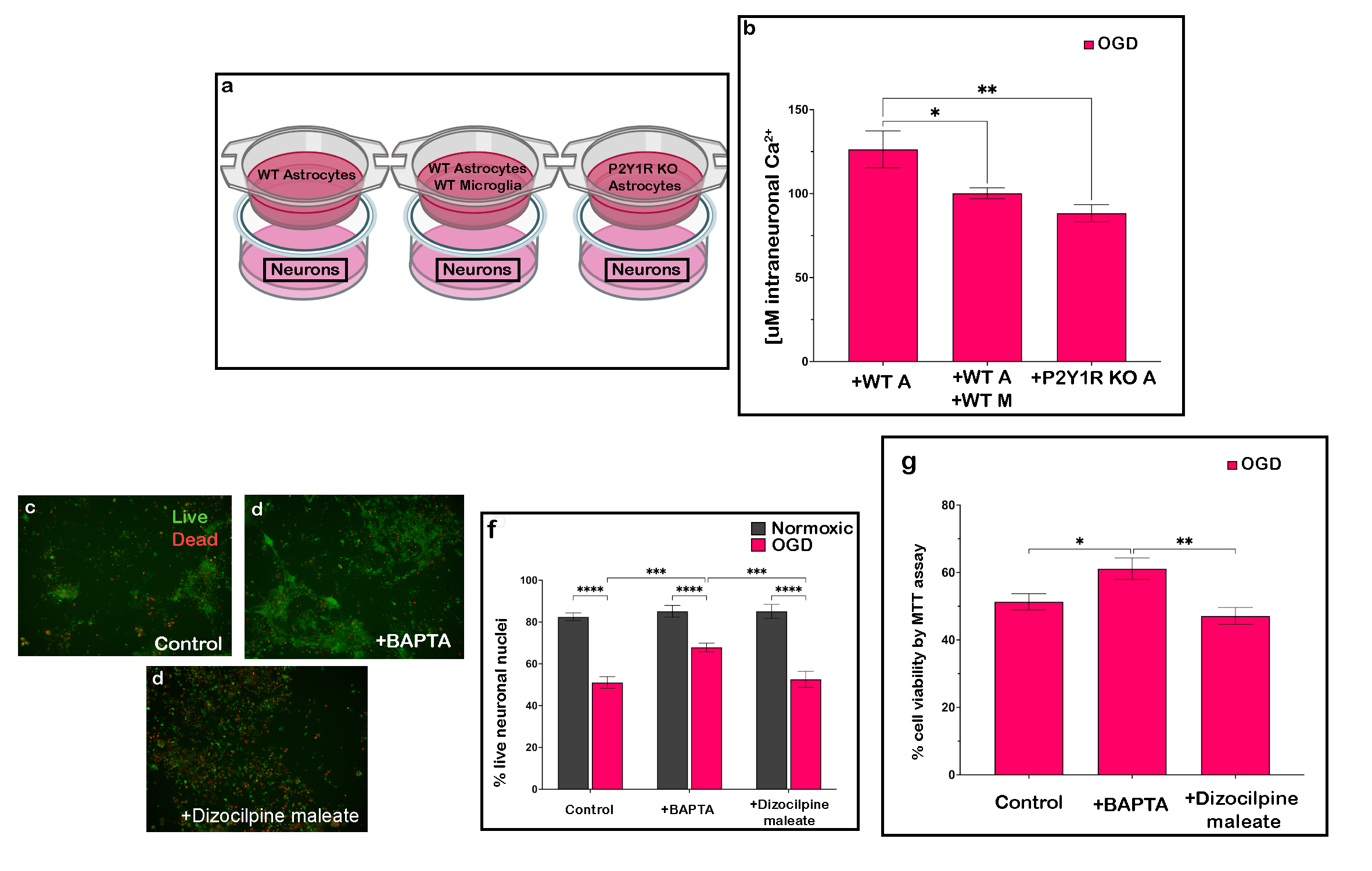

Supplement: Supplementary file 1 [file Data_Sheet_1.ZIP › Suppl Figures/Suppl Figure 4.jpg]

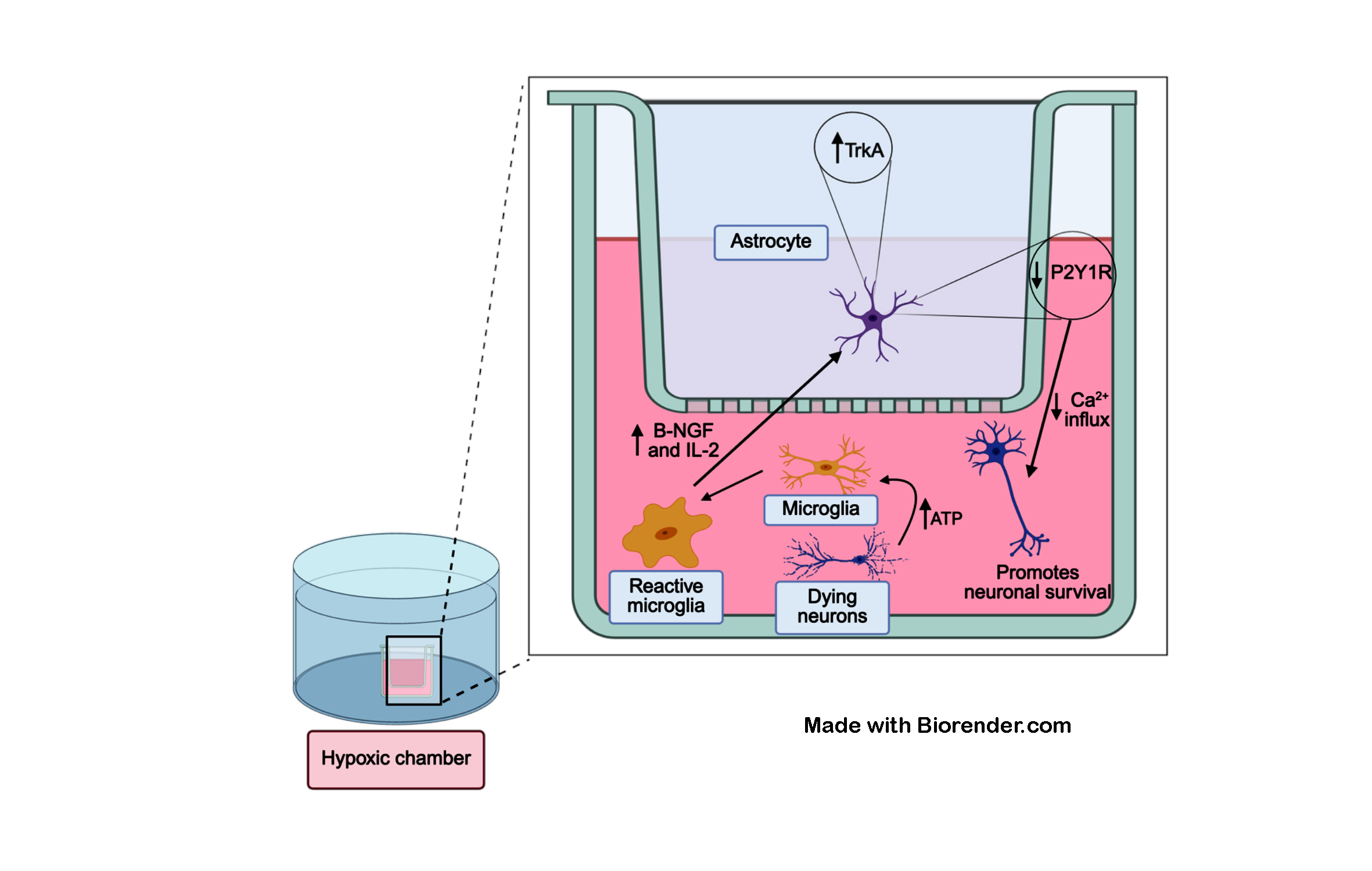

Supplement: Supplementary file 1 [file Data_Sheet_1.ZIP › Suppl Figures/Suppl Figure 5.jpg]

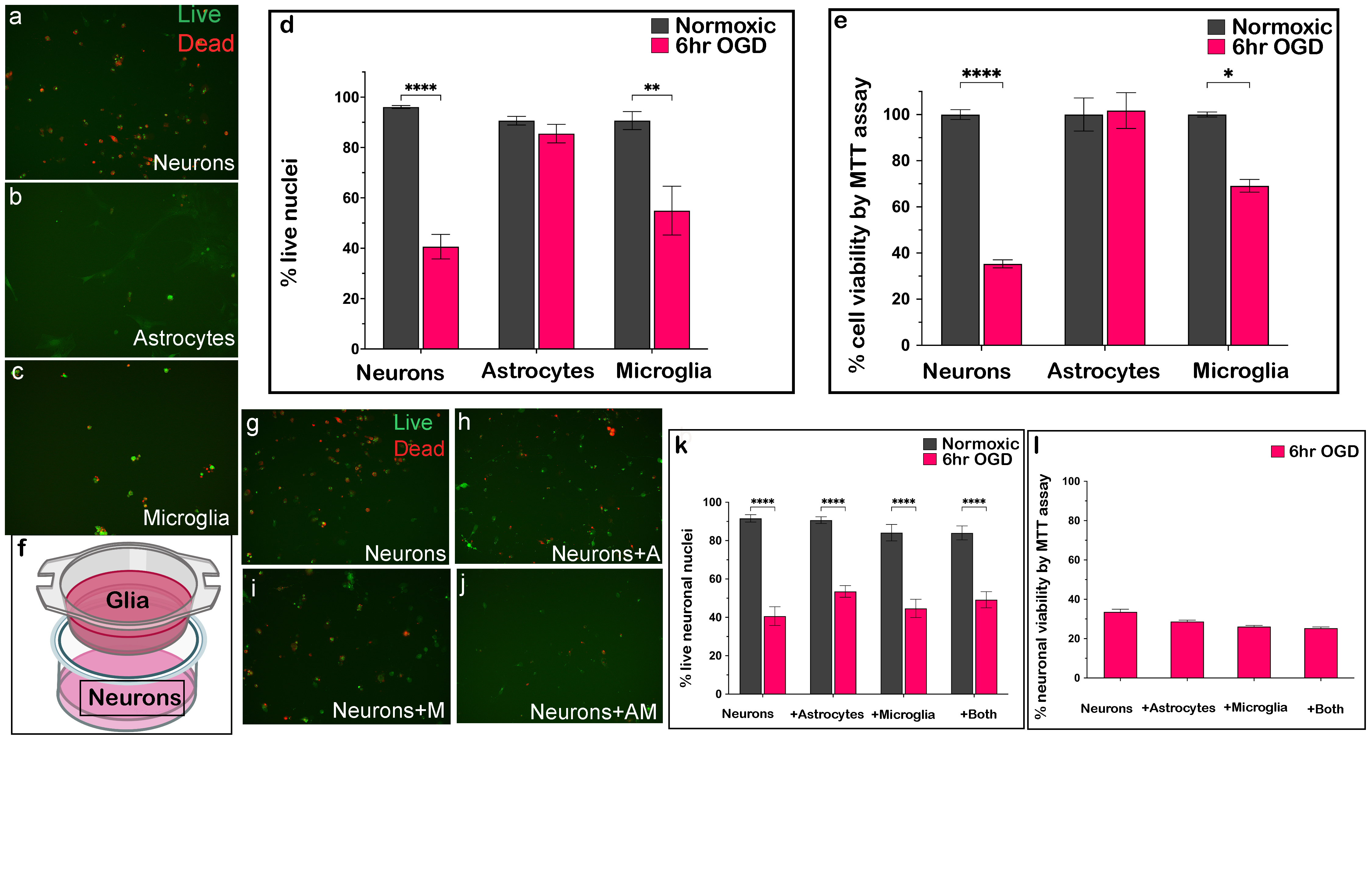

Supplement: Supplementary file 1 [file Data_Sheet_1.ZIP › Suppl Figures/Suppl Figure 1.jpg]

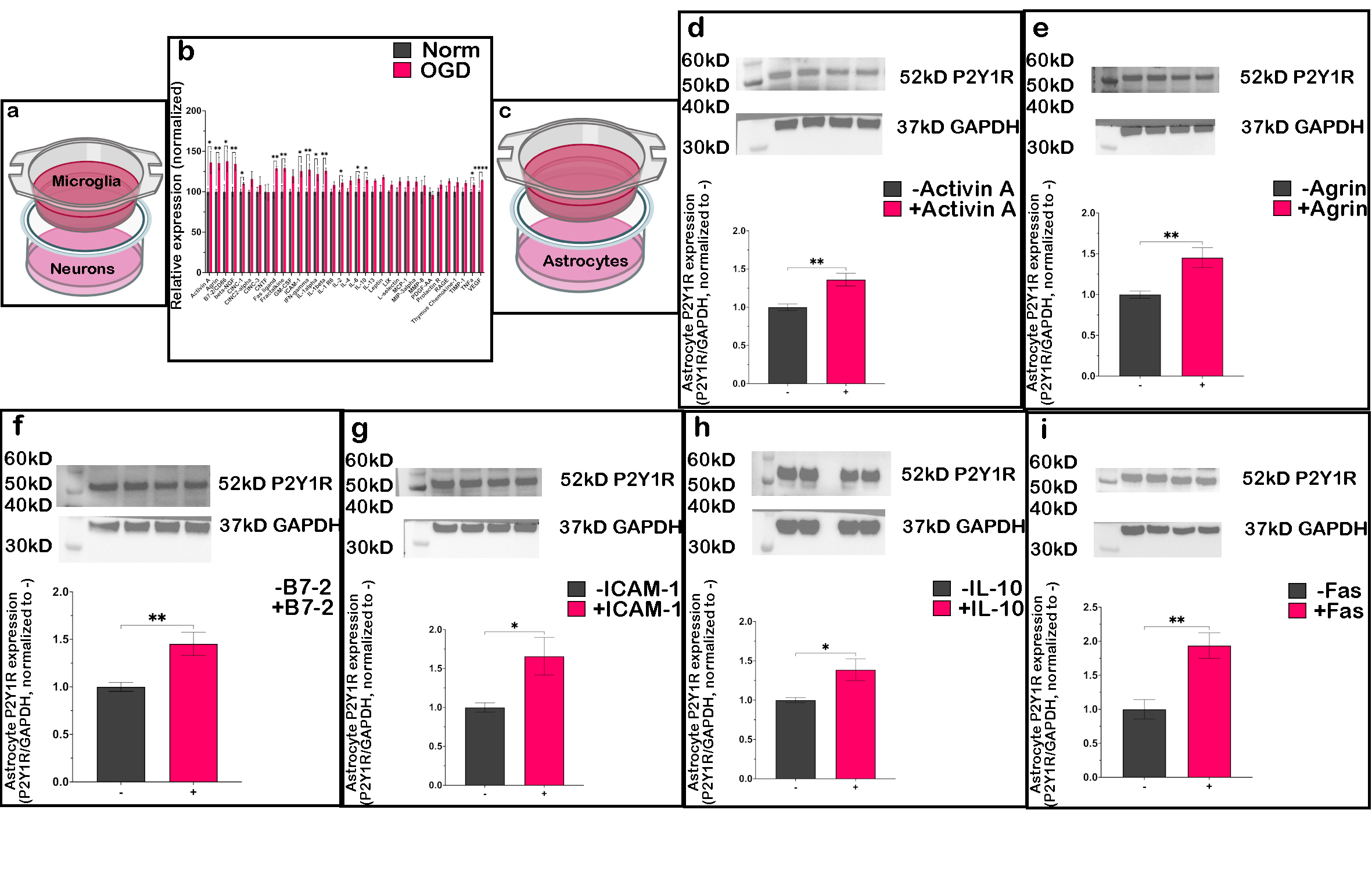

Supplement: Supplementary file 1 [file Data_Sheet_1.ZIP › Suppl Figures/Suppl Figure 2.jpg]

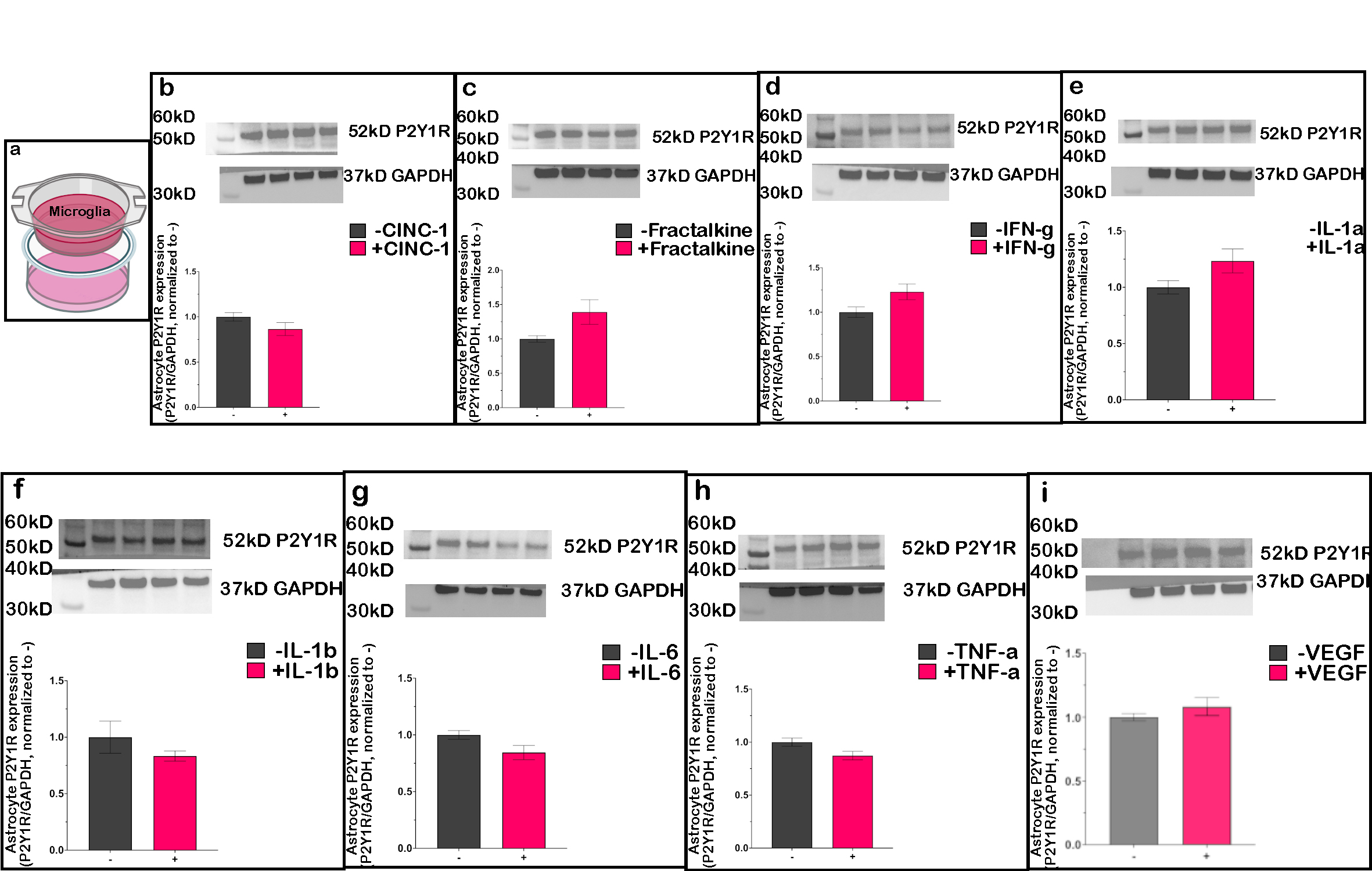

Supplement: Supplementary file 1 [file Data_Sheet_1.ZIP › Suppl Figures/Suppl Figure 3.jpg]
